# I Have Had Enough: When and How Customer Mistreatment Leads to Coworker Undermining

**DOI:** 10.3389/fpsyg.2022.629901

**Published:** 2022-05-09

**Authors:** Zhou Huilian, Muhammad Waqas, Farzan Yahya, Usman Ahmad Qadri, Fatima Zahid

**Affiliations:** ^1^School of Management, Jiangsu University, Zhenjiang, China; ^2^Department of Business Administration, Institute of Southern Punjab, Multan, Pakistan; ^3^Faculty of Business and Management, Universiti Sultan Zainal Abidin, Kuala Terengganu, Malaysia

**Keywords:** customer mistreatment, revenge desire, coworker undermining, social exchange theory, China

## Abstract

Service workers are more prone to experience customer mistreatment because of their frequent interactions with them. Hence, it compels them to the level where their performance is compromised. Employees who face customer mistreatment feel ill-treated and develop the desire for revenge. Based on the social exchange and displaced revenge perspective, this study examined the relationship between customer mistreatment and coworker undermining, and individual-level resource-based moderator service rule commitment (SRC) for this relationship. An analysis of time-lagged, dyadic data (*81 supervisors* and *410 subordinates*) from the Chinese service industry confirmed that customer mistreatment significantly predicted coworker undermining. In addition, in support of the resource perspective, employees’ SRC effectively restricts an effect of customer mistreatment on coworker undermining. Finally, this study contributes to the customer mistreatment and coworker undermining literature by highlighting their relationship. This study also shows the importance of SRC in restraining the adverse effects of customer mistreatment.

## Introduction

The world around us is growing at a fast-paced mode. Economies, markets, trends, fads, and even the people around us are changing. These changes around the globe have psychoanalytically impacted individuals’ behavior. Customers are not exceptional; they are becoming short-tempered, and the more aware they are of the things around, the more consciously they tend to speculate. Customer mistreatment refers to the immoral and unfair treatment of service employees by customers (Wang et al., 2011). Service workers are more prone to experience customer mistreatment because of their frequent interactions with customers (Han et al., 2016). Moreover it is regarded that customer mistreatment is an umbrella concept encircling the vast array of low-quality interactive treatment that employees receive from their customers in the course of service interactions (Bies, 2001; Wang et al., 2011; Koopmann et al., 2015). From throwing hot water on flight attendants to abusing sales staff, numerous cases are recorded daily in-service spaces. Hence, they compels them to the level where their performance is compromised (Koopmann et al., 2015; Van Jaarsveld et al., 2015). Studies show that customers are the most strenuous sources of negativity during service interactions (Yagil, 2017) and that quality service is vital for organizational success, but on instances most employees go through customer mistreatment. Gremler and Gwinner (2000) and Liao and Chuang (2004) accepted that attracting new customers and maintaining customer loyalty is a critical element, so the service organization must provide high-quality services. Employees in service industries are often subject to yelling, swearing, disdainful looks, and unreasonable demands at the hands of their customers. Unlike other forms of interpersonal mistreatment at work, customer mistreatment necessarily comes from outsiders. Customer mistreatment is much more common than insider abuse (Grandey et al., 2007; Sliter et al., 2011). While mistreatment by insiders (e.g., management coercive and ostracization) appears to take place at a low base rate (Bennett and Robinson, 2000), consumer mistreatment is pervasive (Yagil, 2008; Van Jaarsveld et al., 2015). According to Yue (2016), customer mistreatment induces a wide range of consequences, including both psychological and behavioral.

Employees subjected to customer mistreatment treat customers poorly (Van Jaarsveld et al., 2010; Wang et al., 2011), feel burned out (Dormann and Zapf, 2004; Sliter et al., 2010), and withdraw from their work (Grandey et al., 2004; Van Jaarsveld et al., 2015). It has also been observed that employees who are mistreated by customers perform poorly, feel angry and psychologically distressed (Skarlicki et al., 2008; Sliter et al., 2012). In accordance to this, this study incorporates the social exchange theory (SET) that is regarded as one of the oldest theories in the domains of social behavior where social interaction is regarded as an exchange process (Honeycutt, 1981). After employees encounter customer mistreatment, they feel ill-treated and develop the desire for revenge. In doing so, they usually tend to sabotage the customer in an attempt to feel even which, in particular, is a severe violation of the “service rule” or the “script of required behaviors” mandating that customers receive professional, friendly, and customer treatment (Solomon et al., 1985; Skarlicki et al., 2008). The fact that employees possess power over customers does not mean that power obsession should drop in dealings with customers. According to Stuckless and Goranson (1992), revenge is to give the avenger liberation from sore or painful emotions. Still, employees are expected to deliver excellent service despite just suffering customer mistreatment (Van Jaarsveld et al., 2015), which makes them direct their revenge desire away from customers. Displaced revenge is considered a retributive reaction toward some prior mistreatment that is shown against an uninvolved target (Sjöström and Gollwitzer, 2015). In this case, we state that victims of customer mistreatment displace their revenge desire to the nearest possible entity, which could be coworkers, thus they displace their revenge desire toward coworkers and undermine them in an attempt to feel even.

As reported by Yue (2016), customer mistreatment has negative consequences because it divests employees’ resources which they need to deliver high-quality services, but not all employees act in the same way. Research has shown that service rules predict employees’ emotional displays and service delivery (Brotheridge and Grandey, 2002). According to Walsh (2019), employees vary in the level of service commitment they exhibit within any organization. This tends to have a different impact on employees when they experience any kind of customer mistreatment. This makes service rule commitment (SRC) a boundary condition that bounds frontline service employees to engage in emotional labor as part of their job requirements (Walsh, 2019). This impacts how employees treat customers because the level of commitment affects significantly on the way employees respond to adverse events happening around them.

This study aims to contribute to the service literature in various ways. Primarily, it is the first attempt to comprehensively investigate the relationship between two contemporary challenges in the service sector: customer mistreatment and coworker undermining. Interestingly, the theorization and examination of customer mistreatment as an antecedent of coworker undermining behavior is neglected in the literature. This study is the first attempt to hypothesize customer mistreatment as a precursor of coworker undermining behavior. Secondly, coworker undermining behavior is presumably viewed as a threat to employees and the organization at large (Anderson et al., 2004; Duffy et al., 2012) and, as such, we seek to investigate whether the desire for revenge mediates the relationship between customer mistreatment causing employees’ coworker undermining behavior. The third contribution of our study is to examine whether SRC moderates the relations between customer mistreatment, desire for revenge, and employees’ coworker undermining behavior, by either constraining or encouraging the emergence of the desire for revenge and the enactment of revenge against coworkers by undermining them. Therefore, our study not only highlights customer mistreatment as an antecedent of coworker undermining, the mechanism through which it occurs, and the moderating role of SRC, but also helps managers effectively manage those employees who are prone to customer mistreatment. Managers with such insights will be better prepared to handle customer mistreatment.

**FIGURE 1:**
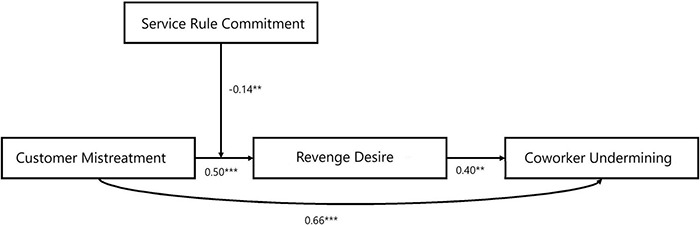
Results of moderated-mediation model. **p < 0.01; ***p < 0.001.

Based on the intuition from SET and the displaced revenge literature, the hypotheses are developed, i.e., customer mistreatment causes coworker undermining, whereas revenge desire explains this relationship. Moreover, we also theorize that SRC weakens the positive association between customer mistreatment and revenge desire. The theoretical contribution, practical implications, and recommendations for future researchers are also discussed in details. The proposed theoretical moderated mediation model is presented in Figure 1.

## Literature and Hypothesis Development

### Customer Mistreatment, Revenge Desire, and Coworker Undermining Through the Lens of Social Exchange Theory

Social exchange theory is traced as “one of the oldest theories of social behavior”—any interaction between individuals is an exchange of resources (Homans, 1958, p. 597). These exchanged resources may not only be tangible, such as goods or money, but could also be intangible, such as social amenities or friendship. The basic assumption of SET is that parties enter into and maintain relationships with the expectation that doing so will be rewarding (Homans, 1958; Blau, 1960). Blau’s (1960) contribution to SET was his economic and social exchange contrasts. He argued that “the basic and essential difference is that undefined responsibilities are a part of social exchange” (p. 93). Lyons and Scott (2012) concluded in a significant extension to the theory about social exchange as “the amount of assistance and harm earned by a certain staff member should be related to the degree to which the employee is helping and harming. The actions shared between employees should also be equal, so that support, but no harm, is related to receiving help and harm, but not helping, is related to harm” (p. 268). A variety of constructs contribute to an unpleasant work environment and behavior. While these principles are applicable to different theoretical models, they are universal in the SET test (Glomb and Liao, 2003; Cohen-Charash and Mueller, 2007; El Akremi et al., 2010) which include deviant behaviors (Robinson and Bennett, 1995), abuse in the workplace (Bowling and Beehr, 2006), and other related causes. Social exchange theorists also argued that the principal drivers of these effects are positive and negative, but the effects are moderated by reciprocity beliefs (Cropanzano et al., 2017).

Following SET, we posit that there is a social exchange relationship between customers and employees, which is very vital and delicate and, as such, must be treated with the utmost tutelage. Once the relationship of social exchange is generated, the manner in which people treat one another is affected. According to Cropanzano et al. (2017), SET treats poor conduct as a reciprocal response to an unfortunate initiating action. Customer mistreatment is the low-quality interpersonal treatment that employees receive from their customers (Bies, 2001); such as a customer treating an employee with disrespect and in an aggressive or unreasonable manner. Customer mistreatment leads to numerous undesired outcomes including the well-known service sabotage (Skarlicki et al., 2008; Wang et al., 2011), work stress (Grandey et al., 2004), and high level of employee negative emotions (Rupp et al., 2008). It breaches the fundamental norms of a good social exchange relationship, hence the service worker feels hurt and develops the desire for revenge. Revenge, which has frequently been cited as the main cause of human aggression (Stuckless and Goranson, 1992) is defined as “basic human impulse and a powerful motivator of social behavior” (Bradfield and Aquino, 1999, p. 17). Philosophers were initially with the conception of revenge being an evil, illogical, and monotonous action that arises in the heat of the moment (Stuckless and Goranson, 1992). In that sense, revenge comes from visceral factors, which overlook rational measures and make people out of control (Loewenstein, 1996; Potegal and Novaco, 2010). In contrast, recent opinions have highlighted the moral and rational nature of the behavior (Tripp and Bies, 2010). Researchers argued in this context that revenge is a logical and moral justification for injustice, aimed at mitigating an error or avoiding future injustice. In this sense, revenge, therefore, acts as a guardian of the social structure and social order (Tripp and Bies, 2010). According to Bies and Tripp (1996), individuals who perceive unfair treatment attempt to seek revenge, which is often manifested in counterproductive work behavior (CWB). It also serves as a protective function for the individual as it restores the psychological balance by providing relief to the victim from all the suffering and psychological pain endured after the transgression, maintains the sense of self-worth, and restores the power balance between the victim and the transgressor (Gollwitzer et al., 2011; Grobbink et al., 2015). Though employees seek revenge from those customers who mistreat them; at the same time employees are required to keep their emotions under control to meet organizationally mandated display rules in the course of service encounters (Grandey, 2000; Diefendorff and Richard, 2003) which can be especially challenging after experiencing customer mistreatment (Goldberg and Grandey, 2007). Studies revealed that customer satisfaction and loyalty are directly affected by the actions and behaviors of service workers (Liao and Chuang, 2004, 2007; Schneider et al., 2005), which in turn affect the profitability and growth of organizations (Reichheld and Sasser, 1990; Anderson and Fornell, 2000; Anderson et al., 2004). With all these, mistreated employees who seek justice divert their revenge desire away from customers. In doing so, the ultimate option left within the organization left is the coworker, so they (mistreated employees) divert the desire for revenge (displaced revenge) toward their coworkers and undermine them.

Undermining, in simple terms, is behavior intended to hinder the designed target over time. Duffy et al. (2002) defined social undermining in the workplace setting as behaviors intended to hinder a colleague’s ability to establish and continue a positive interpersonal relationship, work-related success, and satisfactory repute. It intentionally makes others feel incompetent, talk behind one’s back, and make gossip. A previous work has demonstrated that social undermining can harm organizations by lowering productivity and adversely affecting the emotional states of others (Ruehlman and Wolchik, 1988; Vinokur and Van Ryn, 1993; Vinokur et al., 1996; Westman and Vinokur, 1998; Duffy et al., 2002). This study positions that employees exhibiting undermining behavior toward their coworkers might be as a result of customer mistreatment. Service workers being treated unfairly (with aggression or disrespect) by customers is most likely going to put an employee in a state of hostility or in a bad mood for the rest of the day. Employees in such a state might show behaviors which would undermine their coworkers at the workplace. In this case, customer mistreatment will lead to coworker undermining.

Summarizing the above debate, we state that being mistreated by customers, employees feel hurt and want to payback or get even (revenge) with the customer, but they are unable to take revenge on customers because customer service norms prohibit them from doing so. Employees who feel hurt and victims of injustice by being mistreated by customers will then have to hold back their urge on customers and, in turn, divert the revenge toward their coworkers and undermine them.

H1: *Revenge desire mediates the positive relationship between customer mistreatment and coworker undermining.*

### Moderating Effect of Service Rule Commitment

Another focus of this study is to understand the boundary condition, which may strengthen or weaken the within-person impact of customer mistreatment on coworker undermining. Considering this, our study conceptualized customer mistreatment as a failure of social norms between service employees and customers, leading to undesired outcomes. To control such outcomes, this study incorporates SRC as a potential moderator, which limits coworker undermining behavior through revenge desire. Moreover, a resource-based approach was used to conceptualize customer service engagement in addition to adopting the emotion-based justice viewpoint proposed by Skarlicki et al. (2008) and, as a result, it has been made possible to explore both the emotion-based intervention mechanism and the resource-based mechanisms underlying customer mistreatment—the desire for revenge and customer mistreatment—that undermine linkages. Specifically, a resource-based moderator (SRC) has made a substantial contribution in building up an understanding of customer mistreatment-coworker undermining relationship.

Service rule commitment has been documented as “an employee’s commitment to organizational service rules” (Goldberg and Grandey, 2007, p. 321). It has also been considered as a motivational factor, which shows the extent to which an employee is committed to service goals and push her/himself toward consistently conforming to the assigned service rules even if the situation is not favorable. In other words, it shows the extent to which an employee shows the importance of task-related goals through his/her behavior. Diefendorff et al. (2005) defined it as a person’s intention to extend effort toward exhibiting organizationally desired emotions, to persist in displaying these emotions over time, and not to desolate the display rules under tough situations. They hypothesized display rules as goals that workers strive for over time and across varying situations. The strength of commitment to rules leads employees to choose things that are not necessarily in their best interest, but that trend does not always have a bad impact on their behavior. Deducing from findings in the goal-setting literature (e.g., Locke and Latham, 1990), it has been documented that individuals must be committed to display rules for them to have an impact on behavior. Specifically, we state that employees being committed to service rules will treat customers in a professional manner. Such employees will also intend to exert efforts toward consistently conforming to service rules. Thus, when faced with customer mistreatment, employees with higher SRC are more likely to invest effort in regulating their job-related emotions and behaviors, which, in turn, leads to a lower likelihood of developing a desire for revenge and *vice versa*.

H2a: *SRC will moderate the direct positive relationship between customer mistreatment and revenge desire such that the relationship will be weaker when SRC is high*.

H2b: *SRC will moderate the indirect positive relationship between customer mistreatment and coworker undermining such that the relationship will be weaker when SRC is high*.

## Methodology

### Participants

We collected the data from Chinese employees working in a cell phone company. Specifically, we collected data from 17 offices located in the Jiangsu, Zhejiang, and Henan province. We asked the human resource officials of the targeted offices to distribute the designed survey and also requested them to encourage their employees for maximum participation. Following the nature of the study, only front desk employees who frequently interact with customers were contacted. To control the potential effect of the common bias method, three different surveys were designed. Each survey was distributed at a 2-week gap. We designed Survey 1 to gather respondent demographic information, customer mistreatment, and SRC. Survey 2 was designed to collect information on employees’ revenge desire, whereas Survey 3 was designed to collect information from respondents’ supervisors about their subordinates’ undermining behavior toward his/her colleagues. Overall, 111 supervisors and 493 subordinates took part in this study. However, we received a total of 94 supervisor-administered surveys with a response rate of 85%, and 449 subordinate-administered surveys with a response rate of 91%. Finally, a total of 81 supervisors and 410 subordinates with an average group size of 5,062 participated in this study.

In the study sample, 60% (255) of the employees were men, 40% (170) were women, 65% (275) were in the age limit of 31–36 years, and 68% (290) were at least university graduates with bachelor’s degrees. The majority of employees, 67% (285), had 5–8 years of experience in the service industry.

### Common Method Bias

Although multisource and multi-wave data have the ability to reduce common method variance (CMV) bias, the measurement scales are self-reported, requiring further statistical testing. Since the nature of measurement scales were self-reported. To address this issue, we employed Harman’s one-factor test (Podsakoff et al., 2003). The results showed that the first factor accounted for 36.41% of the total variance and all four factors together produced 72.60% of the total variance. Similarly, in the confirmatory factor analysis (CFA), the common latent factor (CLF) value is equal to 0.612 for all factors at a significant *t*-value. The CMV is the square of that value, 0.6122 = 0.3745. Therefore, the CLF technique suggests there is no significant common method bias (CMB) in these data as the calculated variance (37.45%) is less than the threshold limit of 50%.

#### Measures

The parameters used in this research were authentically created in English. Following the practice of “double-blinded principle” (Brislin, 1980), we used the conventional “translate-back translate” method to convert the English survey into Chinese, and this method was applied to reinforce the reliability and validity of the measures. We requested two Chinese bilingual professors to do the “translate-back-translate” process independently, and then 42 subordinates of nine supervisors (not part of our sample) were requested to do the pretest and give constructive feedback for Chinese survey modification (Aryee and Chen, 2006).

#### Customer Mistreatment

We followed Baranik et al. (2017) and measured customer mistreatment using a 18-item scale developed by Song et al. (2018). Sample items include, “Customers spoke aggressively to you,” and “Customers made demands that you could not deliver.” The responses were recorded on a 5-point Likert scale (1 = never and 5 = always). The alpha reliability was 0.869.

#### Revenge Desire

Following Hongbo et al. (2020b), revenge desire was measured using a five-item scale developed by Jones (2009). The sample items include, “I intend to settle the score with my customers,” and “I plan on getting even with my customers.” The responses were recorded on a five-point Likert scale (1 = strongly disagree and 5 = strongly agree). The alpha reliability of the scale was 0.869.

#### Coworker Undermining

Coworker undermining was measured by a five-item scale developed by Kammeyer-Mueller et al. (2013). Supervisors were asked to indicate the extent to which their subordinates are involved in social undermining behavior at the individual level. Sample items include “Criticizes his/her colleagues,” and “Acts in an unpleasant or angry manner toward others.” Responses were anchored on a five-point Likert scale (1 = to no extent and 5 = to a great extent), where higher scores indicate stronger employee involvement in undermining behavior. Cronbach’s alpha for the scale was 0.922.

#### Service Rule Commitment

We used Wang et al.’s (2011) five-item scale to measure SRC. The scale was originally adopted from Gosserand and Diefendorff (2005). The sample items include “When serving customers, it is hard to take these service rules or not” and “When serving customers, I am committed to conforming to my company’s customer’s service rule.” The respondents were asked to rate the extent to which they agree with the statement (0 = strongly disagree and 5 = strongly agree). Cronbach’s alpha for the scale was 0.870.

#### Control Variables

As suggested by previous studies (Hongbo et al., 2019, 2020b), respondents’ demographics could influence the hypothesized relationship. To avoid model misspecification issues, we controlled for respondents’ age, gender, education, and their experience in the service industry. These demographics have been found to influence corresponding behaviors and social comparison emotions (Tse et al., 2018). Specially, the age and gender of employees are explicitly controlled by previous studies to reduce the biases associated with demographic differences (Eissa and Wyland, 2018) especially related to perceptions of social interaction (Lakey and Cassady, 1990). Undermining is relevant in the context of age because managers have the most power to act on their negative stereotypes about older employees (Ferris et al., 1985). Similarly, women react more proactively against coworker mistreatment or to gain reinclusion following ostracism (Williams and Sommer, 1997; Robinson et al., 2014). Higher education level and longer job tenure are thought to be negatively related to workplace deviance or unethical behavior (Appelbaum et al., 2007; Robinson et al., 2014), thus these individuals are less likely to undermine their coworkers.

## Results

### Descriptive Statistics

Descriptive statistics of all variables are provided in Table 1, including mean, standard deviation (SD), intercorrelation, and Cronbach’s alpha values. In Table 1, the alpha value for each construct above 0.70 is considered acceptable, as suggested by van Griethuijsen et al. (2015). In addition, Table 1 shows that there were the significant and positive correlations between customer mistreatment is positively related to coworker undermining (*r* = 0.188, *p* < 0.01) and SRC (*r* = 0.155, *p* < 0.01). Revenge desire also correlated positively with coworker undermining (*r* = 0.435, *p* < 0.01), SRC (*r* = 0.495, *p* < 0.01), and customers mistreatment (*r* = 0.314, *p* < 0.01). Overall, there are no unexpected results in the correlation matrix.

**TABLE 1:** Inter-correlations, descriptive statistics, and estimated reliabilities among the latent variables.

Variables	*M*	*SD*	Skewness	Kurtosis	Alpha	AVE	MSV	1	2	3	4	5	6	7	8
1.Gender^a^	1.376	0.485	0.516	–1.743	–	–	–	–							
2. Age^b^	1.498	0.724	1.406	1.486	–	–	–	0.024	–						
3. Education^c^	3.605	1.162	–0.609	–0.484	–	–	–	–0.417**	–0.123*	–					
4. Experience^d^	1.778	1.061	1.057	–0.308	–	–	–	–0.056	0.115*	–0.020	–				
5.Coworker undermining	3.654	1.251	–0.757	–0.470	0.922	0.667	0.446	0.027	0.019	0.026	0.059	**(0.817)**			
6.Service rule commitment	3.536	1.293	–0.803	–0.488	0.869	0.689	0.446	0.005	0.002	0.067	0.106*	0.622**	**(0.830)**		
7.Customer’s mistreatment	3.019	1.191	0.014	–1.301	0.869	0.689	0.284	–0.012	–0.035	0.009	0.069	0.188**	0.155**	**(0.713)**	
8.Revenge desire	3.635	1.173	–0.622	–0.929	0.869	0.689	0.284	–0.011	–0.007	0.052	0.082	0.435**	0.495**	0.314**	**(0.830)**

*N = 410 employees; M, Mean; SD, standard deviation; CR, composite reliability; AVE, average variance extracted; MSV, maximum shared variance. Significant at:*

*p < 0.05, **p < 0.01; figures in parentheses are alpha internal consistency reliabilities.

*^a^Gender: 1 = Male, 2 = Female.*

*^b^Age: 1 = 18–24 years, 2 = 25–30 years, 3 = 31–36 years, 4 = over 37 years.*

*^c^Education = 1 = college education, 2 = university graduate, 3 = master/upper education, 4 = others.*

*^d^Experience in service industry: 1 = less than 1 year, 2 = 1–4 years, 3 = 5–8 years, 4 = more than 9 years.*

*Alpha values are indicated in bold.*

In addition, to enhance the validity of the statistical conclusion, the data were screened for outliers using Mahalanobis and Cook’s distance. No observations with extreme values were flagged. Furthermore, homogeneity was ensured using scatter plot and normality with the histogram. The histogram shows that the values were symmetrical and bell-shaped around the mean while the scatter plot show no unequal variability. Lastly, multicollinearity was examined using the variance inflation factor (VIF). None of the VIF values were greater than 1.377, indicating no issue of multicollinearity. Furthermore, this study applied the CFA *via* AMOS to assess the convergent and discriminant validity of the measurement. According to Truong and McColl (2011), regression weights should be equal to or greater than 0.5 for better results. Therefore, the study items’ factor loadings under 0.50 were rejected and were not included in the analysis. For instance, items like, “When serving customers, it is hard to take these service rules or not” are dropped from the analysis due to factor loadings less than the threshold limit. Through the CFA, 26 out of the 32 questions were extracted from the research instrument and 6 questions were dropped due to regression weights less than 0.50, as recommended by Hair et al. (2010). In addition, the measurement model of the study provided the numerous model-fit indexes: χ^2^/df = 318.71/119 = 2.678 < 3.0, “goodness-of-fit index” (GFI) = 0.918, “root mean square error of approximation” (RMSEA) = 0.062, “adjusted GFI” (AGFI) = 0.882, “comparative fit index” (CFI) = 0.959, and “non-normed fit index” (NNFI) = 0.937. As shown in Table 1, the AVE values are greater than the MSV (i.e., discriminant validity), and the AVE of each construct is greater than 0.5 (i.e., convergent validity), which confirms that the measurement model of the study does not have discriminant and convergent validity issues.

### Analytical Approach

Although the study respondents were working in different institutional settings, the subordinates in the same office report to the same supervisor. Thus, the results of ordinary least squares (OLS) regression could produce invalid test statistics or biased standard error (SE) estimations. Prior to testing our empirical model, we calculated the intraclass coefficient 1 (ICC1, variance between supervisors) and intraclass coefficient 2 (ICC2, means’ stability of the supervisors) to evaluate the suitable level of analysis. The ICC1s for customer mistreatment, revenge desire, coworker undermining, and SRC were 0.10, 0.08, 0.13, 0.22, and 0.07 while ICC2s were 0.08, 0.27, 0.10, 0.33, and 0.25, respectively. All these coefficient values are below Cicchetti, 1994 acceptable range of 0.7, which allows us to use the multilevel method.

To further validate our results, we estimated a corrected *F*-statistic of which all are significant, and no value decreased by 0.10. Consistent with Kenny (1995) that individual-level analysis can be estimated when ICCs are below 0.3, we analyze all variables in our model at the individual level.

Many researchers like Borau et al. (2015) have suggested the bootstrapping method (e.g., Process Macro) to test mediation, moderation, and mediated moderation effects over other approaches, such as the causal inference approach, Sobal test, or the Baron and Kenny’s causal step approach. This is because the bootstrapping procedure method is more reliable and more robust for testing moderation and mediation relationships (Borau et al., 2015). Therefore, we used a 5,000-bootstrap sample to assess the conditional indirect effect using a 95% of confidence interval (CI) for the lower and upper limits of the mediated moderation effect (i.e., CI must exclude 0 to be significant), as suggested by Hayes (2017). In line with prior studies (e.g., De Clercq et al., 2021), model 4 of “PROCESS macro” is utilized for the mediation Hypothesis (H1) while model 7 is used for the moderated mediation model (see Tariq and Weng (2018), Hongbo et al. (2019, 2020a,2020b)).

### Test of Mediation

Mediation test is presented in Table 2. Customer mistreatment is positively associated with revenge desire (β = 0.335, *t* = 6.545, *p* < 0.001). Revenge desire is also positively associated with coworker undermining (β = 0.376, *t* = 08.76, *p* < 0.001). Table 2 also indicates significant positive indirect effects of customer mistreatment on coworker undermining *via* revenge desire (β = 0.129, LLCI = 0.085, and ULCI = 0.183). We further perform a Sobel test with a bootstrapped 95% CI to conduct a normal theory test for the indirect effects of customer mistreatment on coworker undermining through revenge desire (Sobel *z* = 5.311, *p* < 0.001). Consequently, our results confirm that the positive association between customer mistreatment and coworker undermining is mediated by revenge desire. Thus, H1 is supported and accepted.

**TABLE 2:** Results of mediation analysis.

Antecedents	Revenge desire						Coworker undermining					
	*B*	*SE*	*T*	*LLCI*	*ULCI*	*R* ^2^	*B*	*SE*	*t*	*LLCI*	*ULCI*	*R* ^2^
						0.105***			0.194***
Constant	2.060	0.419	4.905***	1.234	2.885		1.798	0.373	4.826***	1.066	2.530	
Customers’ mistreatment	0.335	0.051	6.545***	0.235	0.436		0.057	0.047	1.223	–0.035	0.148	
Revenge desire	–	–	–	–	–		0.376	0.043	8.762***	0.291	0.460	
**Control variables**												
Gender	0.006	0.085	0.075	–0.161	0.174		0.038	0.074	0.519	–0.106	0.183	
Age	0.067	0.058	1.150	–0.047	0.181		0.026	0.050	0.508	–0.073	0.124	
Education	0.076	0.058	1.307	–0.038	0.189		0.023	0.050	0.471	–0.075	0.122	
Experience in service industry	0.057	0.139	0.409	–0.216	0.329		0.106	0.119	0.890	–0.128	0.341	

**Predicator**			**Effect**		**SE**			**LLCI**			**ULCI**	

Direct effects			0.057		0.047							
Customers’ mistreatment on coworker undermining			0.056		0.046			–0.034			0.148	
**Indirect effect**												
Customers’ mistreatment on coworker undermining via revenge desire			0.129		0.024			0.085			0.183	
**Total effect**												
Customers’ mistreatment on coworker undermining			0.185		0.048			0.091			0.279	
Normal theory tests for indirect effect			**Effect**		**SE**						**Z**	
Customers’ mistreatment on coworker undermining via revenge desire			0.129		0.024						5.311***	

Results of total, direct, indirect, and normal theory effects. N = 410; Significant at: ***p < 0.001. LLCI, Lower limit confidence intervals at 95%; ULCI, Upper limit confidence intervals at 95%.

### Test of the Moderated Mediation Model

**FIGURE 2:**
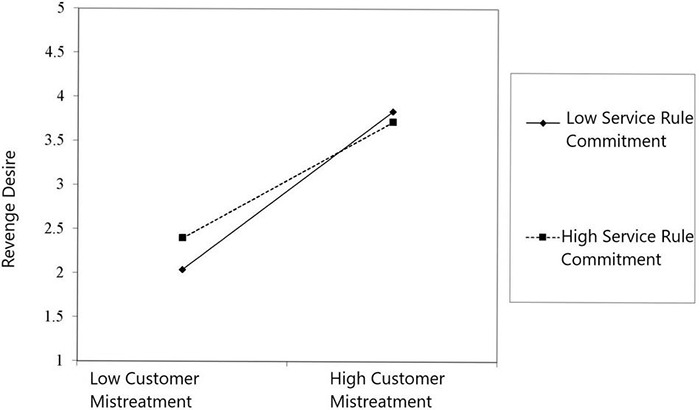
Interactive effect of customer mistreatment and service rule commitment (SRC) on revenge desire.

The findings of the moderated mediation model are presented in Table 3. Similar to the result of the simple mediation analyses, we found that customer mistreatment is positively associated with revenge desire (β = 0.601, *t* = 3.981, *p* < 0.001). Revenge desire is also positively correlated with coworker undermining (β = 0.376, *t* = 8.762, *p* < 0.001). The interaction term between customer mistreatment and SRC is negative and significant (β = -0.019, *t* = -2.353, *p* < 0.001), as shown in Table 3. The second hypothesis (H2a) is supported by the results as SRC moderates the positive relationship between customer mistreatment and revenge desire, such that the positive relationship is weaker when SRC is high. The interaction term is plotted in a graph to further support this hypothesis (see Figure 2). The underlying control variable shows no substantial effect on the main findings, except for the education level effect on revenge desire. As expected, the higher the educational level of the employee, the less likely he/she is to involve in unethical or deviant work behaviors (Appelbaum et al., 2007).

**TABLE 3:** Results of the moderated-mediation model analysis.

Antecedents	Revenge desire						Coworker undermining					
	*B*	*SE*	*T*	*LLCI*	*ULCI*	*R* ^2^	*B*	*SE*	*t*	*LLCI*	*ULCI*	*R* ^2^
						0.312***						0.194***
Constant	–0.088	0.574	–0.154***	–1.216	1.040		1.798	0.372	4.826***	1.0566	2.530	
Customers’ mistreatment	0.601	0.151	3.981***	0.304	0.898		0.057	0.047	1.223	–0.035	0.148	
Revenge desire	–	–	–	–	–		0.376	0.043	8.762***	0.291	0.460	
Service rule commitment (SRC)	0.720	0.115	6.248***	0.494	0.947		–	–	–	–	–	
Customers’ mistreatment × SRC	–0.091	0.039	–2.353	–0.166	–0.015		–	–	–	–	–	
**Control variables**												
Gender	0.0215	0.122	0.176	–0.219	0.262		0.106	0.119	0.890	–0.128	0.341	
Age	0.012	0.075	0.155	–0.136	0.159		0.038	0.074	0.519	–0.106	0.183	
Education	0.032	0.051	0.624	–0.069	0.133		0.026	0.050	0.508	–0.073	0.124	
Experience in service industry	0.019	0.051	0.363	–0.082	0.119		0.024	0.050	0.470	–0.075	0.1220	

N = 410 employees; LLCI, Lower limit of the 95% confidence interval; ULCI, Upper limit of 95% confidence interval; ***p < 0.001.

Finally, to check the presence of mediated moderation in the study, we estimated CIs for the conditional indirect relationship between customer mistreatment and coworker underpinning at different values of SRC (H2a). In Table 4, the results showed the indirect effect of customer mistreatment on coworker undermining *via* revenge desire is weak when SRC is high (β = − 0.034, LLCI = −0.064, ULCI = − 0.005). Likewise, Table 4 shows growing effect sizes at decreasing levels of the SRC: from 0.144 at 1 SD below the mean, 0.102 at the mean, and 0.059 1 SD above the mean. Therefore, we accept H2a.

**TABLE 4:** Results of conditional indirect effects of customer mistreatment on coworker undermining at different values of service rule commitment (SRC).

	Effect size	SE	LLCI	ULCI
Conditional indirect effect (via Revenge Desire) at the different values of Service Rule Commitment; (model 7, Process Macro)^a^				
–1 SD	0.144	0.030	0.088	0.206
Mean	0.102	0.021	0.064	0.144
+ 1SD	0.059	0.025	0.012	0.111
Index of moderated Mediation	–0.034	0.015	–0.064	–0.005

*N = 410; SE, standard error; LLCI, lower-limit of confidence interval; ULCI, upper-limit of confidence interval.*

*^a^Bootstrap analyses based on 5,000 samples.*

## Discussion

One of the essential constituents of the service sector is the quality of interaction between employees and customers. The service sector heavily relies upon the nature of exchange that happens between customers and employees (Doucet et al., 2016). As the frequency of interaction with customers is higher in the service sector than that anywhere else, employees are likely to experience mistreatment (Han et al., 2016). Not only this, but the mundane and overemphasized concept that is mere a cliché that “Customer is the king” gives them the edge and make them feel entitled to mistreat employees. Employees subjected to mistreatment struggle to enact key service behaviors (Shao and Skarlicki, 2014; Baranik et al., 2017), conceal their emotions (Grandey et al., 2004), become upset and angry (e.g., Rupp and Spencer, 2006), and feel burned out (Greenbaum et al., 2014). Employees who are mistreated by customers and who wish to preserve fairness are more likely to react in ways that are covert or undetectable by customers or other organizational participants, such as delivering suboptimal quality service or not being especially polite when talking to customers because of these peculiar characteristics of the service setting (Madupalli and Poddar, 2014). In this way, workers (who have experienced consumer mistreatment) and their peers (who have observed this mistreatment) sense a mental fatigue that may weaken them (Shao and Skarlicki, 2014).

It is observed that the positive connection between customer mistreatment and coworker undermining in the face of revenge desire is weaker when SRC is high. In summary, we argue that all of our proposed hypotheses are proven through the empirical findings and are also well aligned with previous studies related to such negative workplace behaviors. In the model of organizational vengeance, these results are consistent with previous research indicating that workers who are mistreated by customers will ignite the drive for vengeance, i.e., revenge desire against the cause of mistreatment if they perceive that justice will not be preserved in any way (Van Jaarsveld et al., 2010). However, being part of the service industry, employees are compelled to hide their inner feelings and behave in a socially acceptable manner, i.e., they do not revert back to ill-mannered customers, rather they exhibit self-control and behave ethically. In addition, the results also supported the negative association between SRC–revenge desire relationship and also the buffering role of SRC between customer mistreatment–revenge desire relationships. These outcomes are also aligned with the previous studies like the negative relationship of SRC–sabotage against customers and the moderating role of SRC between customer mistreatment–sabotage against customers (Wang et al., 2011). The study, promisingly, has shed light on how customer mistreatment negatively influences employee emotions and behavior. Through the findings of the study, it is clearly evident that there exists a positive relationship between customer’s mistreatment and coworker undermining behavior, whereas revenge desire mediates this relation.

The study has been a vital addition in theoretical nuances as the current examination is one of the first to look at the association between the mistreatment of organizational workers by customers and coworkers in the presence of SRC as a moderator. We also developed a resource-based approach to conceptualize customer service engagement, in addition to adopting the emotion-based justice viewpoint proposed by Skarlicki et al. (2008). As a result, we have been able to explore both the emotion-based intervention mechanism and the resource-based mechanisms underlying customer mistreatment—the desire for revenge and customer mistreatment—that undermine linkages. Specifically, a resource-based moderator (SRC) has made a substantial contribution in building up understanding about customer mistreatment—employee retaliation and customer mistreatment—coworker weakening associations.

The service sector plays a major role in the economy now, and one way to stabilize its performance is to impart such procedural and smooth working among service workers and customers so that undesired encounters can be avoided. As the frequency of employee–customer interaction is higher in the service sector than anywhere else, mistreatment events are more common, which leads to employees’ undesired behavior (Han et al., 2016). To avoid such undesired behaviors, this study shows the potential value of resource-based factor, i.e., SRC, which concludes that employees’ undesired behavior could be avoided by standardizing a set of rules and training to inculcate higher SRC. Moreover, understanding the possible factors, which underlie coworker undermining, is also an important step in trying to manage this undesired behavior. Normally, when undermining is detected, managers relate it to employees’ personality, which narrows their strategies to control the undermining issue. Meanwhile, this study shows that employees’ undesired behavior is not always due to their own personality, there are some reasons beyond their control. For that, this study considered customer mistreatment as an antecedent of employees’ coworker undermining behavior. One of the strategies for organizations to manage mistreatment is to track such customers who violate interpersonal ethics and take steps to (a) prepare employees for potential ill-mannered customers, (b) provide employees with the discretion to terminate mistreating customers. In addition, a zero-level tolerance policy for mistreating customers is likely to signal employees that the company cares about the respect of its employees. Another strategy is to train employees to deal better with ill-mannered customers.

In summary, this study shows that employees engage in coworker undermining as a reaction to mistreatment received from customers. The coworker undermining incidents arising from customer mistreatment, however, are moderated by SRC, i.e., the relationship between customer mistreatment and coworker undermining is weak for those employees who are highly committed to service rules.

### Limitations and Future Recommendations

We suggest that our study opens up new dimensions in the customer mistreatment literature, which encourages future researchers to explore this domain further to learn more about it. Despite some interesting findings, this study is also accompanied by some limitations. First, our findings confirm that the positive relationship between customer mistreatment and coworker undermining is mediated by the desire for revenge. This indicates the possibility of some other mechanisms, which may also explain this relationship, such as ego depletion, emotional exhaustion, and psychological distress. Thus, it is recommended that future researchers examine other mechanisms underlying these relationships. Second, this study is limited to the scope of the moderator examined. Specifically, this study focuses on investigating the individual-level factor (SRC), which moderates the relationship between customer mistreatment and the desire for revenge. However, future researchers might extend this study by examining if some unit- or organization-level factors provide additional resources for employees to better deal with customer mistreatment. For instance, customer-focused service climate may encourage service sector employees to stick with the service rule, which ultimately restricts employees’ negative behavior, accordingly, we collected data from 17 offices located in the three different provinces of China. However, we did not examine the influence of potential differences across these offices. We suggest that it is important to consider this difference. Our third limitation is related to methods. Although this study is based on time-lagged multisource data (supervisors and subordinates), one cannot completely control the issue of CMV because most of the key variables were reported by the same respondents, e.g., customer mistreatment, revenge desire, and SRC. Nevertheless, we did our best to minimize the issue of CMV by using multisource data, yet we are afraid that CMV might have affected our findings. For that, we suggest future researchers to seriously consider this issue in their study. We encourage future researchers to use the daily diary method to eliminate the issue of CMV.

Fourth, the study is based on data collected in a single culture (China). There is a possibility that other factors associated with the organization or respondents have shaped the responses such that generalizability is reduced. Future research involving respondents from various cultures is warranted. Fifth, this study is based on a cross-sectional design that limits causal references. This can be a quiet possibility that the employees who undermine coworkers are indeed more unreceptive and thus act in ways that make customers unreceptive too.

## Data Availability Statement

The raw data supporting the conclusions of this article will be made available by the authors, without undue reservation.

## Ethics Statement

The studies involving human participants were reviewed and approved by Ethics Committee at Jiangsu University, China. Written informed consent for participation was not required for this study in accordance with the national legislation and the institutional requirements.

## Author Contributions

All authors equally contributed to conception and design, acquisition of data, analysis and interpretation of data, drafted the article for important intellectual content, approved final version to be published and agreement to be accountable for all aspects of the work in ensuring that questions related to the accuracy and integrity of any part of the work are appropriately investigated and resolved.

## Conflict of Interest

The authors declare that the research was conducted in the absence of any commercial or financial relationships that could be construed as a potential conflict of interest.

## Publisher’s Note

All claims expressed in this article are solely those of the authors and do not necessarily represent those of their affiliated organizations, or those of the publisher, the editors and the reviewers. Any product that may be evaluated in this article, or claim that may be made by its manufacturer, is not guaranteed or endorsed by the publisher.
